# Root‐knot nematodes exploit the catalase‐like effector to manipulate plant reactive oxygen species levels by directly degrading H_2_O_2_

**DOI:** 10.1111/mpp.70000

**Published:** 2024-09-10

**Authors:** Zhaolu Zhu, Dadong Dai, Mengzhuo Zheng, Yiling Shi, Shahid Siddique, Feifan Wang, Shurong Zhang, Chuanshuai Xie, Dexin Bo, Boyan Hu, Yangyang Chen, Donghai Peng, Ming Sun, Jinshui Zheng

**Affiliations:** ^1^ National Key Laboratory of Agricultural Microbiology, College of Life Science and Technology Huazhong Agricultural University Wuhan China; ^2^ Hubei Key Laboratory of Agricultural Bioinformatics, College of Informatics Huazhong Agricultural University Wuhan China; ^3^ Department of Entomology and Nematology University of California Davis California USA

**Keywords:** catalase, CATLe, effector, *Meloidogyne incognita*, reactive oxygen species

## Abstract

Plants produce reactive oxygen species (ROS) upon infection, which typically trigger defence mechanisms and impede pathogen proliferation. Root‐knot nematodes (RKNs, *Meloidogyne* spp.) represent highly detrimental pathogens capable of parasitizing a broad spectrum of crops, resulting in substantial annual agricultural losses. The involvement of ROS in RKN parasitism is well acknowledged. In this study, we identified a novel effector from *Meloidogyne incognita*, named CATLe, that contains a conserved catalase domain, exhibiting potential functions in regulating host ROS levels. Phylogenetic analysis revealed that CATLe is conserved across RKNs. Temporal and spatial expression assays showed that the *CATLe* gene was specifically up‐regulated at the early infection stages and accumulated in the subventral oesophageal gland cells of *M. incognita*. Immunolocalization demonstrated that CATLe was secreted into the giant cells of the host plant during *M. incognita* parasitism. Transient expression of CATLe significantly dampened the flg22‐induced ROS production in *Nicotiana benthamiana*. In planta assays confirmed that *M. incognita* can exploit CATLe to manipulate host ROS levels by directly degrading H_2_O_2_. Additionally, interfering with expression of the *CATLe* gene through double‐stranded RNA soaking and host‐induced gene silencing significantly attenuated *M. incognita* parasitism, highlighting the important role of CATLe. Taken together, our results suggest that RKNs can directly degrade ROS products using a functional catalase, thereby manipulating host ROS levels and facilitating parasitism.

## Graphical abstract

CATLe, a novel secreted effector from root‐knot nematodes, exhibits catalase activity, regulates plant ROS levels by directly catalysing H_2_O_2_ decomposition, suppresses plant immune responses and promotes parasitism.

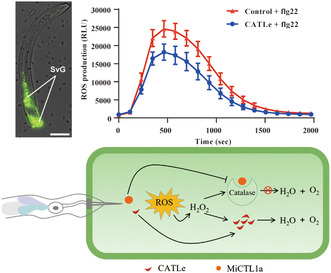

## 1 INTRODUCTION

Polyphagous root‐knot nematodes (RKNs, *Meloidogyne* spp.) are notorious soilborne pests that cause severe agriculture yield losses and are considered an important risk factor for global food security (Forghani & Hajihassani, 2020; Warmerdam et al., 2018). The infective second‐stage juveniles (J2s) of RKNs use a hollow protrusive stylet to penetrate the plant root elongation zone and migrate intercellularly toward the vascular cylinder, where they establish feeding sites and become sedentary (Abad et al., 2008; Nguyen et al., 2018). The feeding sites, composed of five to seven large multinucleate cells known as giant cells, serve as the primary nutrient pool for RKN growth and reproduction (Favery et al., 2020). The establishment of feeding sites, coupled with the proliferation of plant tissue surrounding the giant cells, results in the formation of knot‐like galls, impeding the transport of water and nutrients through the plant roots and ultimately leading to yield losses (Siddique & Grundler, 2018).

The oxidative burst, a hallmark of plant innate immunity characterized by the rapid, massive and transient production of reactive oxygen species (ROS) including H_2_O_2_ and O_2_^−^, plays important roles in plant's resistance against pathogens (Ali et al., 2018; Langebartels et al., 2002; Wu & Ge, 2004; Zhou et al., 2018). ROS can cause DNA oxidative damage, trigger a hypersensitive response (HR) to block pathogen colonization and serve as signalling molecules in plant defence cascades (Wang et al., 2020). Notably, ROS have been identified to play important roles in plant resistance against RKNs; for instance, the induction of increased ROS accumulation in rice resulted in reduced infection by the RKN *Meloidogyne graminicola* (Chavan et al., 2022; Holbein et al., 2016). To withstand the host‐derived ROS stress, RKNs have evolved sophisticated mechanisms to manipulate the ROS metabolism of host plants (Rutter et al., 2022). Previous research has confirmed that using effectors to suppress the host ROS production is an important strategy employed by RKNs. For instance, the effector Mg16820 from *M. graminicola* can interact with dehydration stress‐inducible protein 1 of plants to suppress ROS production (Naalden et al., 2018), while the effector MiPDCD6 from *Meloidogyne incognita* can inhibit ROS accumulation by suppressing salicylic acid‐mediated plant immunity (Kamaruzzaman et al., 2023).

Additionally, evidence indicates that the host ROS scavenging systems are important targets of RKNs. For example, the effector MgMO289 from *M. graminicola* can interact with rice copper metallochaperone heavy metal‐associated plant protein 04 (OsHPP04) to activate the host O_2_^•−^‐scavenging system (Song et al., 2021). Similarly, the effector MjTTL5 from *Meloidogyne javanica* can enhance plant ROS scavenging activities through interaction with ferredoxin of *Arabidopsis* (Lin et al., 2016). In addition, ROS‐scavenging enzymes, such as peroxiredoxins in *M. incognita*, are localized within the nematode's hypodermis, suggesting a role in protecting the nematode's body against ROS (Dubreuil et al., 2011). While several effectors involved in regulating plant ROS have been identified in RKNs, their primary function is to regulate plant ROS levels by interacting with plant proteins or disrupting plant immune signalling pathways. However, whether effector proteins of *M. incognita* can directly target ROS products of host plants needs further investigation.

In this study, a novel effector with catalase activity, named CATLe, was identified from the sedentary endoparasitic nematode *M. incognita*. Our results suggest that CATLe is specifically up‐regulated at the early infection stages and localizes in the subventral oesophageal gland cells of *M. incognita*. Immunolocalization confirmed that CATLe can be secreted into plant giant cells, where it regulates plant ROS levels by directly catalysing the decomposition of H_2_O_2_. Comprehensive functional characterization underscores the critical role of CATLe in facilitating parasitism. These findings provide valuable insights into the mechanism by which *M. incognita* regulates plant ROS levels through effectors.

## 2 RESULTS

### 2.1 Identification of the *M. incognita* catalase‐like effector

**FIGURE 1:**
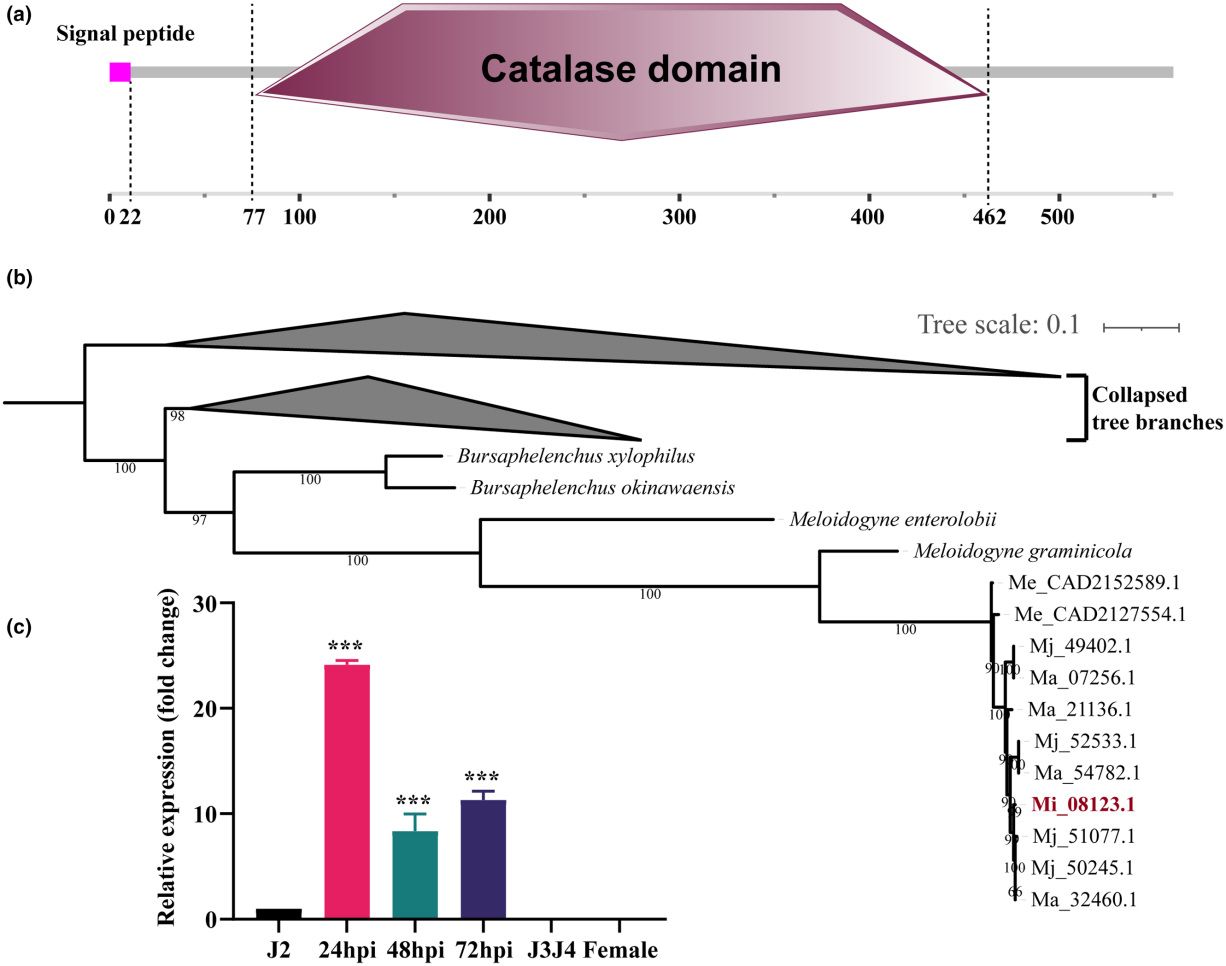
Characterization of the *Meloidogyne incognita* catalase‐like effector. (a) Primary structure of CATLe. (b) Phylogenetic tree of proteins homologous to CATLe based on the maximum‐likelihood method. The grey shapes represent the collapsed tree branches. (c) Reverse transcription‐quantitative PCR validation of relative expression level of *CATLe* in *M. incognita* at different infection stages. The fold‐change values were analysed by the 2^−ΔΔ*C*t^ method. The *GAPDH* gene of *M. incognita* was used as a reference. ***p < 0.001, Student's *t* test. Bars represent mean ± *SD*.

In this study, we identified a protein named CATLe (Mi_08123.1) from the *M. incognita* genome (Genome_assembly_V1) (Dai et al., 2023) that contains a secretory signal peptide and a catalase domain, consists of 560 amino acids and has a calculated molecular weight of 61,803 Da (Figure 1a). Catalase is a core antioxidant enzyme in most organisms; it catalyses the decomposition of H_2_O_2_ into water and molecular oxygen (Baker et al., 2023). Notably, H_2_O_2_ is a major component of ROS, which inhibit pathogen infection and trigger local and systemic defence responses (Dutta et al., 2023). Thus, we speculated that CATLe may exhibit potential functions in regulating host ROS levels during *M. incognita* parasitism.

To explore the presence of CATLe in other plant‐parasitic nematodes, we conducted a genome‐wide homology search and secretory signal peptide prediction. As a result, we identified two proteins (Me_CAD2152589.1 and Me_CAD2127554.1) with a secretory signal peptide and a conserved catalase domain in the *Meloidogyne enterolobii* genome (Koutsovoulos et al., 2020), four proteins (Ma_07256.1, Ma_54782.1, Ma_21136.1 and Ma_32460.1) in the *Meloidogyne arenaria* genome and four proteins (Mj_49402.1, Mj_52533.1, Mj_51077.1 and Mj_50245.1) in the *M. javanica* genome. Multiple sequence alignment showed that these proteins shared high similarities with CATLe (Figure S1). Additionally, a homologue search identified 476 homologous proteins (*E* < 10^−5^) of CATLe in the NR database. Phylogenetic analysis demonstrated that the proteins with a secretory signal peptide and a conserved catalase domain in different RKNs fall into one subgroup (Figure 1b). Quantification of *CATLe* transcripts by reverse transcription‐quantitative PCR (RT‐qPCR) at different infection stages of *M. incognita* confirmed that *CATLe* was strongly up‐regulated at 24, 48 and 72 h post‐infection (hpi) compared to the preparasitic J2s stage and almost no expression was observed at later stages (Figure 1c). These results suggest that CATLe is conserved among RKNs and may play an important role in the early stages of nematode parasitism.

### 2.2 CATLe is expressed in the subventral oesophageal gland and can be secreted into giant cells

**FIGURE 2:**
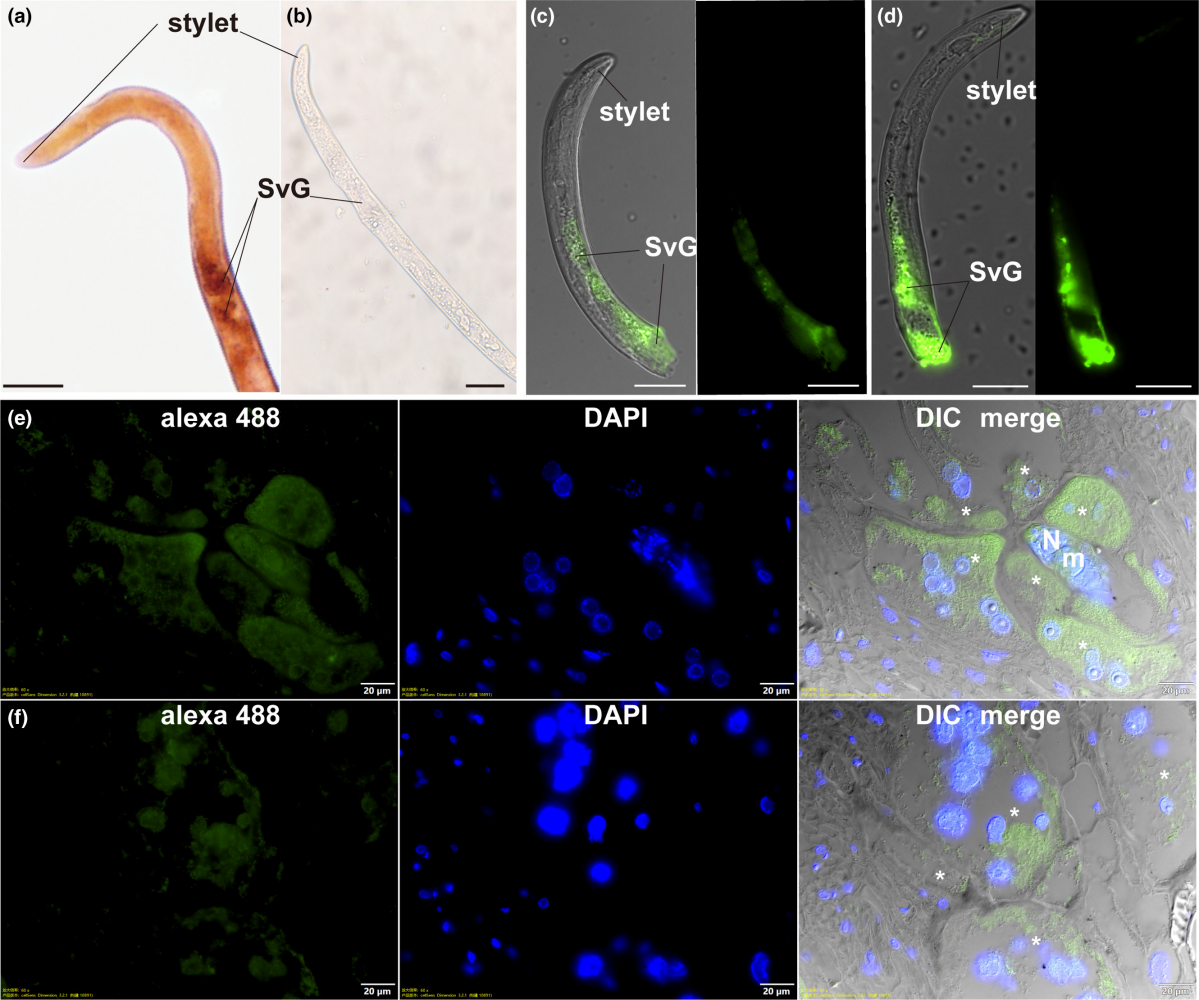
Localization of CATLe. (a) In situ hybridization (ISH) of digoxigenin‐labelled antisense *CATLe* probe to preparasitic *Meloidogyne incognita* second‐stage juveniles (J2s) indicates the transcription of *CATLe* in the subventral oesophageal gland (SvG). Bar = 20 μm. (b) No ISH signal was observed in the *CATLe* sense probe (right). Bar = 20 μm. (c–e) Immunolocalization with the anti‐CATLe antibody showed that the CATLe protein was present in the SvG of preparasitic *M. incognita* J2s. Bar = 20 μm. (e, f) Immunolocalization of the secreted CATLe protein in tomato tissues. CATLe accumulated in the giant cell. The images are shown by merging the differential interference contrast (DIC), DAPI‐stained nuclei and Alexa Fluor 488 fluorescence. N, nematodes; m, metacorpus; *, giant cells, bar = 20 μm.

As the effectors of plant‐parasitic nematodes are mainly secreted by subventral and the dorsal oesophageal gland cells, we performed in situ hybridization to investigate the localization of *CATLe* transcripts in *M. incognita* preparasitic J2s. A strong signal was detected in subventral oesophageal gland (SvG) cells with the digoxigenin‐labelled CATLe probe (Figure 2a), while no signal was observed in preparasitic J2s with negative controls (Figure 2b). Additionally, we generated an antibody to CATLe to investigate the immunolocalization of CATLe in preparasitic J2s. The specificity of the antibody was confirmed through western blot analysis, demonstrating a clear band of the anticipated size in the total proteins extracted from preparasitic J2s (Figure S2). As expected, immunolocalization showed that the CATLe was present in the SvG of preparasitic J2s (Figure 2c,d), which is consistent with the in situ hybridization result. These results suggest that CATLe could potentially act as an effector of *M. incognita*.

To examine whether CATLe is secreted into host plants during *M. incognita* parasitism, the immunolocalization was performed on tomato gall sections. Western blot analysis confirmed the specificity of the antibody, as no signal was observed in samples from uninfected tomato plants (Figure S2). Immunohistochemistry conducted on sections of tomato root galls at 5 days post‐inoculation (dpi) revealed the presence of CATLe within the giant cells (Figure 2e,f). As anticipated, no signal was detected in planta when employing pre‐immune serum (Figure S3). These results confirm that CATLe is an effector that can be secreted into plant cells and is probably functional during *M. incognita* parasitism.

### 2.3 CATLe exhibits catalase activity and can dampen plant ROS production

**FIGURE 3:**
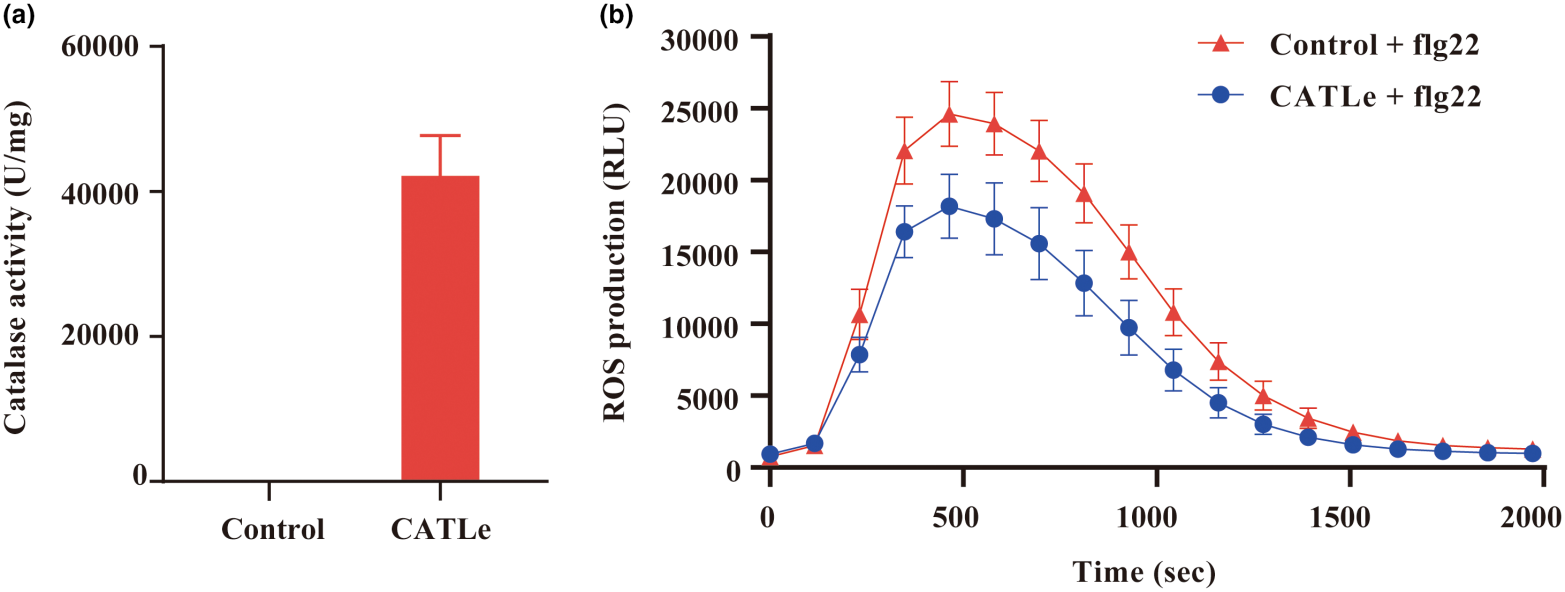
CATLe exhibits catalase activity and suppresses reactive oxygen species (ROS) burst. (a) Catalase activity assay for purified CATLe. The *Escherichia coli* expression strain transformed with the empty vector served as a negative control. Bars represent mean ± *SD*. (b) CATLe suppressed the flg22‐induced ROS production in *Nicotiana benthamiana* leaves. *Agrobacterium tumefaciens* GV3101 carrying CATLe‐coding sequence and red fluorescent protein (mCherry) was infiltrated into 4‐week‐old leaves of *N. benthamiana*. Leaf discs (diameter 4 mm) were collected and incubated with 100 nM flg22 after 48 h infiltration to perform the ROS assay. The curve was drawn by the average values of the relative luminescence unit (RLU). Bars represent mean ± *SD*. Control represents *N. benthamiana* with mCherry infiltration.

To examine whether CATLe is a functional catalase that can catalyse the decomposition of H_2_O_2_, the CATLe‐coding sequence without the signal peptide was cloned and expressed in *Escherichia coli* Rosetta (DE3). Enzyme activity assays for purified CATLe demonstrated that CATLe had an enzyme activity of about 42,500 U/mg (Figure 3a). To examine its catalytic activity in vivo, CATLe (without signal peptide) was transiently expressed in *Nicotiana benthamiana* leaves. Subsequently, we detected the ROS production in *N. benthamiana* leaf discs under flg22 stimulation. A comparison with the negative control showed that ROS production was significantly decreased in *N. benthamiana* leaf discs under the expression of CATLe (Figure 3b). These results suggest that CATLe can efficiently dampen the flg22‐induced ROS production.

### 2.4 CATLe targets plant ROS products by directly degrading H_2_O_2_

**FIGURE 4:**
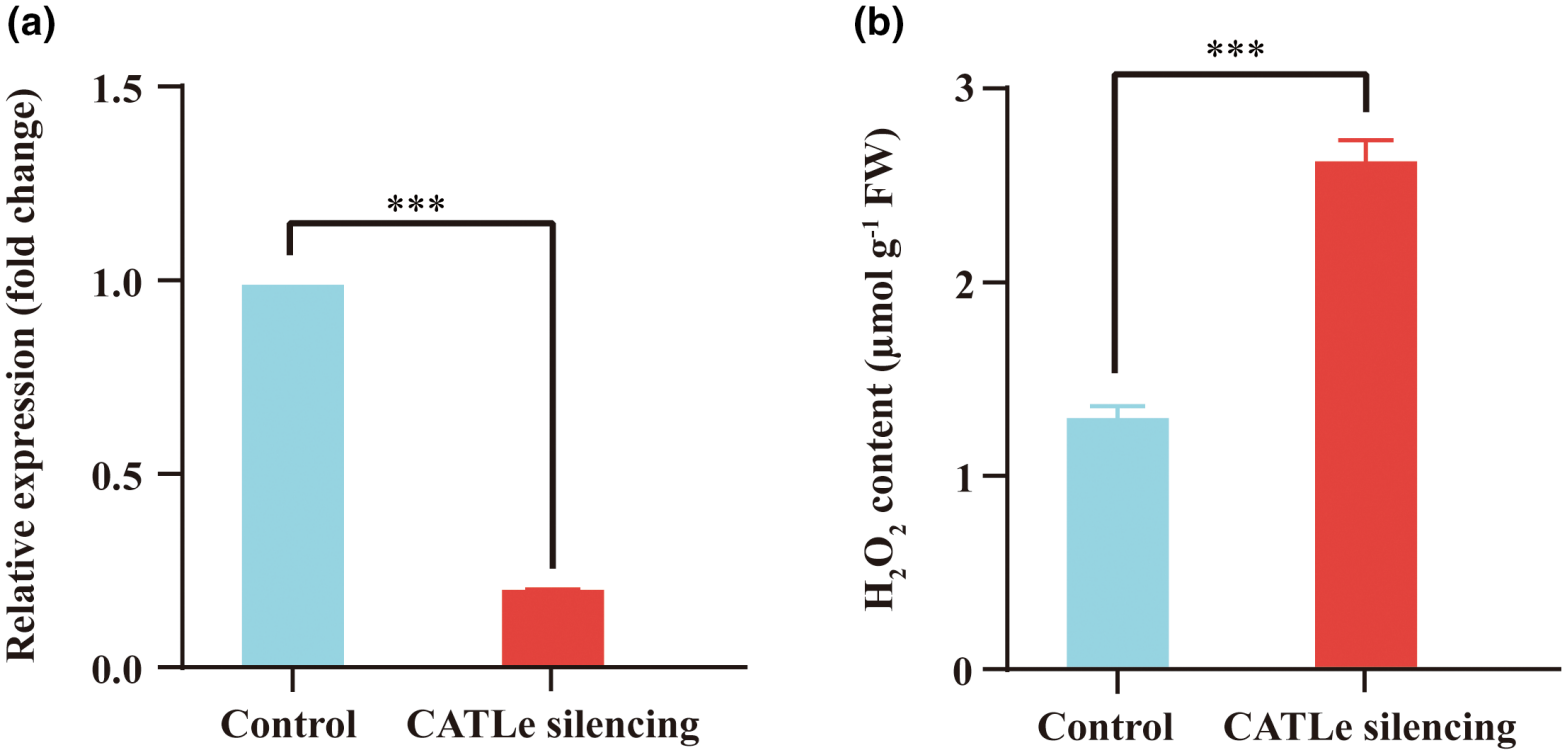
CATLe targets plant reactive oxygen species products by directly degrading H_2_O_2_. (a) Interference efficiency detection of *CATLe* gene by reverse transcription‐quantitative PCR. The fold‐change values were analysed by the 2^−ΔΔ*C*t^ method. The *GAPDH* gene of *Meloidogyne incognita* was used as a reference, ***p < 0.001. (b) CATLe silencing leads to an increased in host H_2_O_2_ accumulation. The H_2_O_2_ content in tomato seed radicles was measured at 24 h post‐inoculation, ***p < 0.001.

To further confirm the role of CATLe in regulating host H_2_O_2_ levels, we silenced the expression of the *CATLe* gene in *M. incognita* preparasitic J2s through soaking with double‐stranded (ds) RNA. A 208 bp fragment of the CATLe‐coding sequence was selected as the interference target and the non‐target green fluorescent protein gene (*gfp*, 287 bp) was used as the negative control. After soaking *M. incognita* preparasitic J2s in dsRNA (CATLe/GFP), RT‐qPCR analysis showed a significant decrease in *CATLe* gene expression compared to the negative control, indicating effective silencing (Figure 4a). Subsequently, we inoculated the dsRNA (CATLe/GFP)‐treated nematodes onto tomato seed radicles. At 24 hpi, we measured the H_2_O_2_ levels in the infected radicles. Our results revealed a significant increase in H_2_O_2_ levels in tomato seed radicles infected by CATLe‐silenced nematodes compared to the negative control (Figure 4b). These results confirm that *M. incognita* employs CATLe to directly degrade plant H_2_O_2_.

### 2.5 CATLe silencing affects *M. incognita* parasitism

**FIGURE 5:**
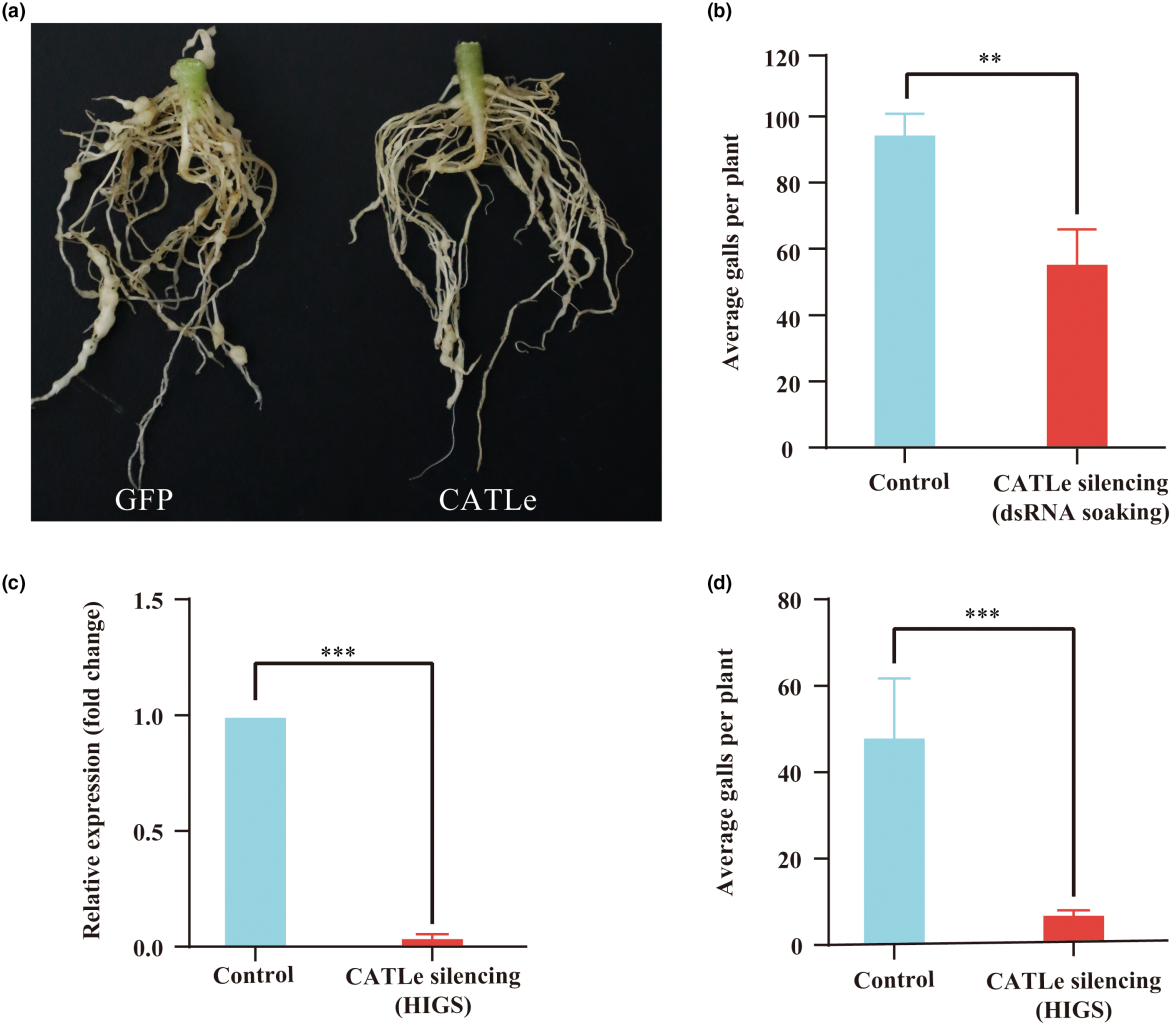
*CATLe* silencing affects *Meloidogyne incognita* parasitism. (a) *CATLe* silencing leads to a decrease in gall size induced by *M. incognita* infection. (b) *CATLe* silencing leads to a decrease in gall number induced by *M. incognita* infection. Four‐week‐old tomato seedlings were inoculated with 300 dsRNA (GFP/CATLe)‐treated preparasitic J2s and galls were counted at 20 days post‐inoculation (dpi). (c) Expression level of *CATLe* gene detected by reverse transcription‐quantitative PCR (RT‐qPCR). Transgenic *Nicotiana benthamiana* lines expressing hairpin dsRNA (GFP/CATLe) were inoculated with 2000 preparasitic J2s. The infected roots were collected at 5 dpi and the RNA was extracted for RT‐qPCR. (d) Host‐induced gene silencing (HIGS) for *CATLe* decreased the gall number induced by *M. incognita* infection in *N. benthamiana*. The transgenic lines expressing hairpin dsRNA (GFP/CATLe) were inoculated with 200 fresh‐hatched preparasitic J2s. The galls were counted at 20 dpi. **p < 0.01, ***p < 0.001, Student's *t* test. Bars represent mean ± *SD*.

To investigate the role of CATLe in *M. incognita* parasitism, we inoculated the dsRNA (CATLe/GFP)‐treated nematodes onto 4‐week‐old tomato seedlings and counted the galls at 20 dpi. We found that silencing the *CATLe* gene led to a significant reduction in both the number and size of galls induced by *M. incognita* infection compared to the negative control (Figure 5a,b). Additionally, we explored the role of CATLe in *M. incognita* parasitism using host‐induced gene silencing (HIGS). Transgenic *Nicotiana tabacum* plants expressing CATLe hairpin dsRNA were generated, with transgenic plants expressing GFP hairpin dsRNA serving as negative controls. After inoculating transgenic plants with nematodes, RT‐qPCR analysis at 5 dpi showed a significant decrease in *CATLe* expression in *M. incognita* compared to negative controls, indicating effective silencing of *CATLe* (Figure 5c). Moreover, there was a notable decrease in the number of galls induced by *M. incognita* (at 20 dpi) in the transgenic plants (Figure 5d). Overall, these results indicate that CATLe plays a crucial role in facilitating *M. incognita* parasitism.

## 3 DISCUSSION

As a principal ROS component, H_2_O_2_ plays a critical role in counteracting pathogen infection by inducing plant‐programmed cell death (Zhang et al., 2014). Moreover, H_2_O_2_ can also mediate downstream immune signalling in plants: some key phytohormones involved in the regulation of plant stress response, including salicylic acid, jasmonates, abscisic acid and ethylene, employ H_2_O_2_ in their signalling cascades in an either upstream or downstream manner (Saxena et al., 2016). In addition, H_2_O_2_ is relatively stable and can diffuse among cellular compartments or cells, which facilitates its signalling functions (Bienert et al., 2007; Henzler & Steudle, 2000). Therefore, increasing attention has been paid to the role of H_2_O_2_ in the interaction between pathogens and plants. For instance, it has been identified to play a critical role in the susceptibility of *Brassica napus* to *Leptosphaeria maculans* (Nováková et al., 2016). PsCAT1, a monofunctional heme‐containing catalase secreted by *Puccinia striiformis*, acts as an important pathogenic factor to facilitate the infection of *P. striiformis* by scavenging host‐derived H_2_O_2_ (Yuan et al., 2021). Similarly, *Blumeria graminis* can also secrete extracellular catalase to clear H_2_O_2_ during infection of barley (Zhang et al., 2004).

**FIGURE 6:**
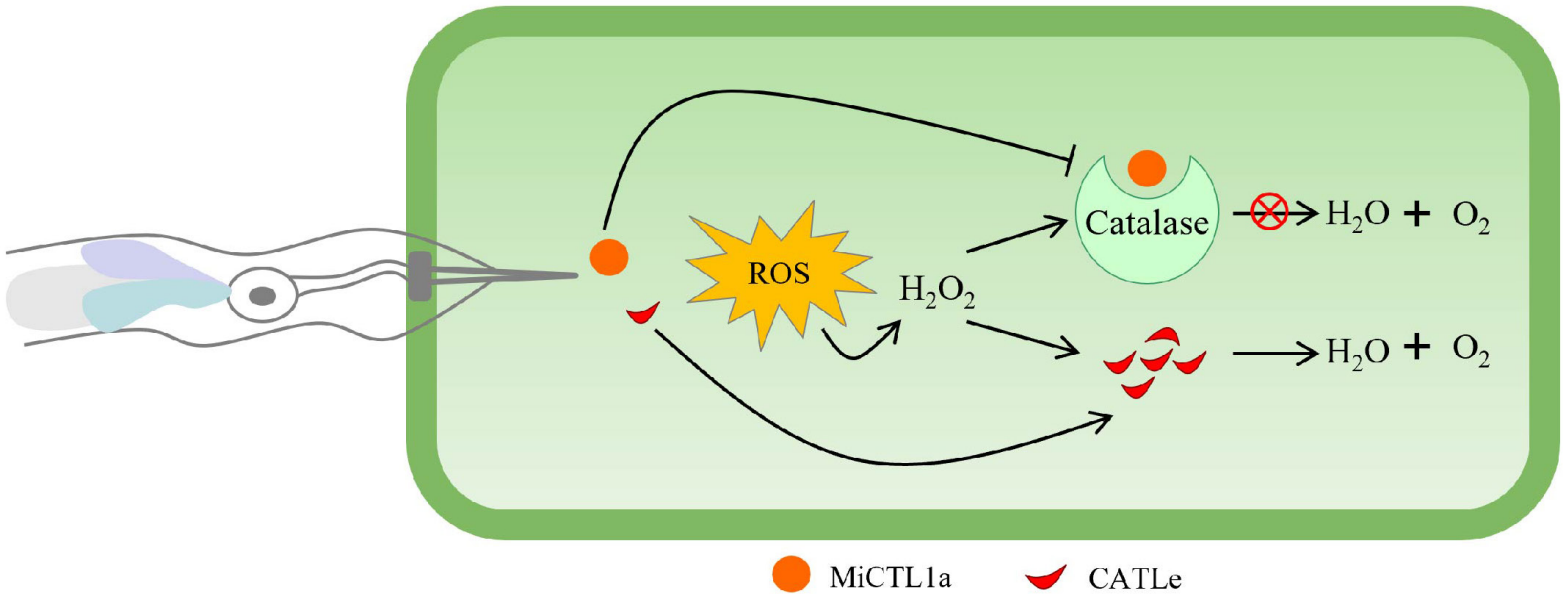
Schematic illustration depicts how *Meloidogyne incognita* uses effectors to manipulate the plant H_2_O_2_ levels during early infection. *M. incognita* secretes MiCTL1a to interact with plant catalase, suppressing its activity and preventing H_2_O_2_ decomposition by plants. *M. incognita* also secretes CATLe to catalyse H_2_O_2_ decomposition, potentially enabling nematodes to autonomously regulate the H_2_O_2_ levels of plants.

In this study, through an investigation into effectors modulating plant ROS levels during the initial infection stages of *M. incognita*, we discovered a novel effector, CATLe, that exhibits catalase activity and is specifically up‐regulated during the early infection stages (Figures 1c and 3a). In vitro and in planta assays confirmed the critical roles of CATLe in suppressing the plant ROS burst and regulating plant H_2_O_2_ levels (Figures 3b and 4b). Interestingly, recent research identified a C‐type lectin (CTL)‐like effector from *M. incognita*, named MiCTL1a, that has been shown to interact with the catalase of plants, inhibiting its activity (Zhao et al., 2021). Through expression pattern analysis and RT‐qPCR validation, we showed that *CATLe* and *MiCTL1a* were simultaneously up‐regulated during early infection stages (Figure 1c; Figure S4). Therefore, based on these findings, we propose that *M. incognita* can use the effector MiCTL1a to interact with plant catalase and reduce its activity, potentially inhibiting H_2_O_2_ decomposition by plants, and subsequently employs the effector CATLe to further degrade plant H_2_O_2_ (Figure 6). This mechanism may enable nematodes to autonomously regulate the H_2_O_2_ levels of plants.

Because RKNs are obligate biotrophic parasites, the manipulation of plant H_2_O_2_ levels implies that successful parasitism of RKNs requires the maintenance of a certain level of H_2_O_2_. There have been increasing studies indicating that H_2_O_2_ is a critical signalling molecule that mediates a variety of biological processes in plants such as cell differentiation and proliferation (Holmström & Finkel, 2014; Huang et al., 2019). The H_2_O_2_ levels have also been demonstrated to play important roles in meristem enlargement, cell expansion and cell wall extensibility (Liszkay et al., 2004; Tsukagoshi et al., 2010). More importantly, previous research has indicated that H_2_O_2_ is also accumulated in the cell wall of giant cells in feeding sites induced by RKNs (Melillo et al., 2006). The formation of feeding sites induced by RKNs involves complex manipulation of host signal transduction to activate the redifferentiation of root cells into hypertrophied and multinucleate giant cells (Medina et al., 2017; Mejias et al., 2021; Noureddine et al., 2022). In this study, silencing the CATLe of *M. incognita* not only altered the plant H_2_O_2_ levels but also resulted in a smaller gall size induced by *M. incognita* (Figures 4b and 5a). These results suggest that the regulation of plant H_2_O_2_ levels is not only important for *M. incognita* to resist host‐derived ROS stress but also for it to induce the giant cells. Together, our research sheds light on the molecular mechanisms in regulating ROS levels employed by *M. incognita* and highlights the critical role of effector CATLe in promoting parasitism.

## 4 EXPERIMENTAL PROCEDURES

### 4.1 Nematode and plant materials

*Meloidogyne incognita* was propagated on glasshouse‐grown tomato (*Solanum lycopersicum* ‘Jinpeng No. 3’) at 25°C under 16 h:8 h, light:dark conditions. *S. lycopersicum* ‘Heinz 1706’ seeds were germinated to collect tomato seed radicles for the in planta assay. *N. benthamiana* was used to perform the ROS burst detection assay. *Nicotiana tabacum* was used to generate the transgenic plants.

### 4.2 Sequence analysis, alignment and phylogenetic tree

CATLe in RKNs was obtained from newly published *Meloidogyne* genomic resources (Genome_assembly_V1) (Dai et al., 2023). Protein domain annotation was performed on SMART (https://smart.embl.de/). The homologous proteins of CATLe were identified from NR database using Diamond BLASTP (v. 2.0.4.142) with *E*  < 10^−5^. Multiple sequence alignment of protein sequences was performed by MUSCLE (v. 3.8.1551) (Edgar, 2004) and trimmed by trimAL (v. 1.4.rev22) (Capella‐Gutiérrez et al., 2009). The trimmed alignments were then used to construct the phylogenetic tree by IQ‐TREE (v. 2.0.3) (Nguyen et al., 2015) with 1000 bootstrap replicates and the LG + R8 model. The phylogenetic tree was visualized using iTOL (Letunic & Bork, 2019).

### 4.3 RNA extraction and quantitative real‐time PCR

The total RNA was extracted using TRIzol (Invitrogen) according to the manufacturer's instructions. Reverse transcription was performed using HiScript III All‐in‐one RT SuperMix Perfect for qPCR (Vazyme). The expression of genes was determined using the Hieff UNICON Universal Blue qPCR SYBR Green Master Mix (YEASEN) by RT‐qPCR. The relative changes in gene expression were determined using the 2^−ΔΔ*C*t^ method.

### 4.4 In situ hybridization and immunolocalization of CATLe

With a simple modification of the previously described method (Jaouannet et al., 2018), the digoxigenin (DIG)‐labelled specific probe was amplified with specific primers (Table S1). In situ hybridization (ISH) was performed on fresh‐hatched preparasitic J2s of *M. incognita* using the MyLab DIG Labeling and Hybridization Detection System‐DIG DNA PCR Labeling Kit (MyLab Corp.) according to the description of the manufacturer. Briefly, the nematodes were fixed with 4% formaldehyde for 24 h and chopped on a glass slide. Proteinase K (1 mg/mL) was used to digest the chopped nematodes for 2 h. The nematode was hybridized with a DIG‐labelled specific probe at 40°C for 16 h and then the hybridization signal was detected with anti‐DIG antibodies and observed under a microscope.

The polyclonal antibody against CATLe was produced by ABclonal Biotech Co. Ltd against a specific peptide (C‐AEYWNELSPVDRQH). The specificity of anti‐CATLe antibodies was confirmed by western blot. The immunolocalization on *M. incognita* preparasitic J2s was performed as previously described (Zhao et al., 2020, 2021). Briefly, the preparasitic J2s of *M. incognita* were used to perform the immunolocalization directly with the anti‐CATLe antibody (1:500) and the Alexa Fluor 488 fluorescence‐conjugated goat anti‐rabbit antibody (1:5000). Nematodes incubated with pre‐immune serum served as a negative control. Results were imaged using confocal microscopy (FV1000; Olympus). To validate the accumulation of CATLe within the giant cells, the root galls were collected at 7 dpi and after paraffin embedding and sectioning, the paraffin was removed using a dewaxing solution (G1128; Wuhan Servicebio Technology). The gall sections were treated with ethanol solutions and incubated in a blocking solution of 1% bovine serum albumen for 30 min. Subsequently, the gall sections were incubated with anti‐CATLe antibody (1:200) at 4°C overnight. Then, after shaking and washing three times on a shaker for 5 min each time to destain them with phosphate‐buffered saline (pH 7.4), the gall sections were treated with the Alexa Fluor 488 fluorescence‐conjugated goat anti‐rabbit antibody (1:400). Finally, shaking and washing three times again, using 4′,6‐diamidino‐2‐phenylindole (DAPI) (Wuhan Servicebio Technology) to stain the cell nuclei and imaged using confocal microscopy. The gall sections incubated with pre‐immune serum served as a negative control.

### 4.5 Heterologous expression of CATLe and enzyme activity measurement

The CATLe‐coding sequence without signal peptide (Mi_08123.1, Mi_assembly_v1) was cloned and inserted into the prokaryotic expression vector pET‐15D. The recombined vector was then transformed into the expression strain *E. coli* Rosetta (DE3). Protein production was induced by adding 0.2 mM isopropyl‐β‐d‐thiogalactopyranoside (IPTG) to the cell culture. After 16 h of expression at 16°C, the His‐CATLe protein was purified using Ni‐agarose column (CWBIO) according to the description of the manufacturer. After purification, the eluted protein was concentrated and desalinized by a 30‐kDa cut‐off Centricon filter (Millipore).

To assess the enzyme activity of CATLe, the protein concentration of CATLe was measured firstly using the BCA Protein Assay Kit (Coolaber) following the instructions of the manufacturer. Then, the enzyme activity of CATLe was measured following the previous descriptions (Zhao et al., 2021) using the Catalase Activity Assay Kit (Beyotime). A catalase activity assay on the purified protein from the same expression strain transformed with the recombinant vector and the empty vector was performed to exclude the possibility of catalase originating from bacteria.

### 4.6 ROS burst detection

The CATLe‐coding sequence without signal peptide (Mi_08123.1, Mi_assembly_v1) was amplified and inserted into the pCAMBIA1300s vector to generate a recombinant vector and then transformed into *Agrobacterium tumefaciens* GV3101. The pCAMBIA1300s vector with mCherry was used as the control. The leaves of 4‐week‐old *N. benthamiana* were infiltrated with recombinant *A*. *tumefaciens* GV3101. The leaf discs (diameter 4 mm) expressing CATLe and mCherry were collected after 48 h of infiltration and incubated with 200 μL sterile water in 96‐well plates in the dark for 16 h. The ROS production of the leaf discs was measured in 100 μL detection buffer (34 μg luminol, 20 μg horseradish peroxidase and 100 nM flg22) using a microplate reader (Victor NIVO 3S; PerkinElmer). All experiments were performed three times and 16 leaf discs were analysed per treatment.

### 4.7 CATLe silencing and measurement of plant H_2_O_2_ content

The region (CDS_683‐891bp_, 208 bp) of CATLe‐coding sequence (Mi_08123.1, Mi_assembly_v1) was selected as the interference target to knock down the expression of *CATLe* using dsRNA soaking for preparasitic J2s. Non‐target green fluorescent protein coding sequence (*gfp*, 287 bp) was used as the negative control. The dsRNA was synthesized using T7 RNAi transcription kit (Vazyme) using specific primers (Table S1) and following the instructions of the manufacturer. A final concentration of dsRNA exceeding 1 μg/μL was obtained. RNAi was performed by soaking about 20,000 preparasitic J2s in 250 μL RNase‐free water containing 1 μg/μL dsRNA (CATLe/GFP) and 1% resorcinol. Three biological replicates were performed. After 24 h of soaking at 25°C, the soaking solution was removed and the dsRNA‐treated preparasitic J2s was recovered in 500 μL RNase‐free water for 2 h. The tomato seeds (*S. lycopersicum* ‘Heinz 1706’) were germinated as described above. The radicles with root hairs were immersed in dsRNA‐treated preparasitic J2s for 10 s and then transferred into the growth substrate (50% soil and 50% sand). The remaining dsRNA‐treated preparasitic J2s were collected to extract the RNA and detect the interference efficiency of RNAi.

After 24 h of infection at 25°C, the radicles were collected with the removal of excess nematodes by running water and used to measure the content of H_2_O_2_ by Hydrogen Peroxide Assay Kit (Beyotime) according to the manufacturer's instructions. Briefly, the radicles were homogenized with the lysis buffer and centrifuged at 12,000 *g* at 4°C for 5 min. The supernatants were then transferred into a 96‐well plate and incubated with hydrogen peroxide detection solution for 30 min at 25°C. The absorbance was detected at 560 nm with a microplate reader (Victor NIVO 3S; PerkinElmer). The content of H_2_O_2_ was calculated according to a standard curve.

### 4.8 Infection assay of *M. incognita*

Infection assays were performed under infection by *CATLe*‐silenced preparasitic J2s using the HIGS and dsRNA‐soaking methods. The dsRNA soaking of preparasitic J2s was performed as described above. Four‐week‐old tomato seedlings (*S. lycopersicum* ‘Heinz 1706’) were inoculated with 300 dsRNA (CATLe/GFP)‐treated preparasitic J2s. Three biological replicates were performed and five tomato seedlings were inoculated per replicate. Galls were counted at 20 dpi. Statistical analysis was performed with Student's *t* test.

For the HIGS method, the sense and antisense sequence of the region (CDS_683–891 bp_, 208 bp) in CATLe‐coding sequence (Mi_08123.1, Mi_assembly_v1) were cloned and inserted into pBWA(V)HS‐RNAi to generate the hairpin vector constructs using specific primers (Table S1). The recombined vectors were then transformed into *A. tumefaciens* GV3101 and a positive colony was used to infect the leaf disc of *N. tabacum*. Transgenic *N. tabacum* plants were selected using 50 mg/L hygromycin. The genomic DNA was extracted from the potential transgenic *N. tabacum* (T_0_) and the transgenic lines were screened by PCR using specific primers (Table S1). The total RNA was isolated from the transgenic lines (T_1_) and the expression level of the dsRNA was detected by RT‐qPCR using specific primers (Table S1).

Transgenic *N. tabacum* expressing hairpin dsRNA of *gfp* was used as a control (generated by another research laboratory). The transgenic lines expressing hairpin dsRNA (GFP/CATLe) were inoculated with 2000 preparasitic J2s. The infected roots were collected at 5 dpi and the RNA was extracted for RT‐qPCR to measure the expression level of CATLe in nematodes. Three transgenic lines with a high expression level of hairpin dsRNA (CATLe) were inoculated with 200 preparasitic J2s and five plants were inoculated per line. The galls were counted at 20 dpi and statistical analysis was performed with Student's *t*test.

## CONFLICT OF INTEREST STATEMENT

The authors declare no competing interests.

## Supporting information


Figure S1.

Figure S2.

Figure S3.

Figure S4.

Table S1.

## Data Availability

The data that supports the findings of this study are available in the supplementary material of this article.
