# Supplementary material for: Root‐knot nematodes exploit the catalase‐like effector to manipulate plant reactive oxygen species levels by directly degrading H2O2
Source: Mol Plant Pathol. 2024 Sep 10;25(9):e70000. doi: 10.1111/mpp.70000 (PMC11386320; doi:10.1111/mpp.70000)
Supplement: Supplementary file 1 — Figure S1. [file MPP-25-e70000-s002.docx]

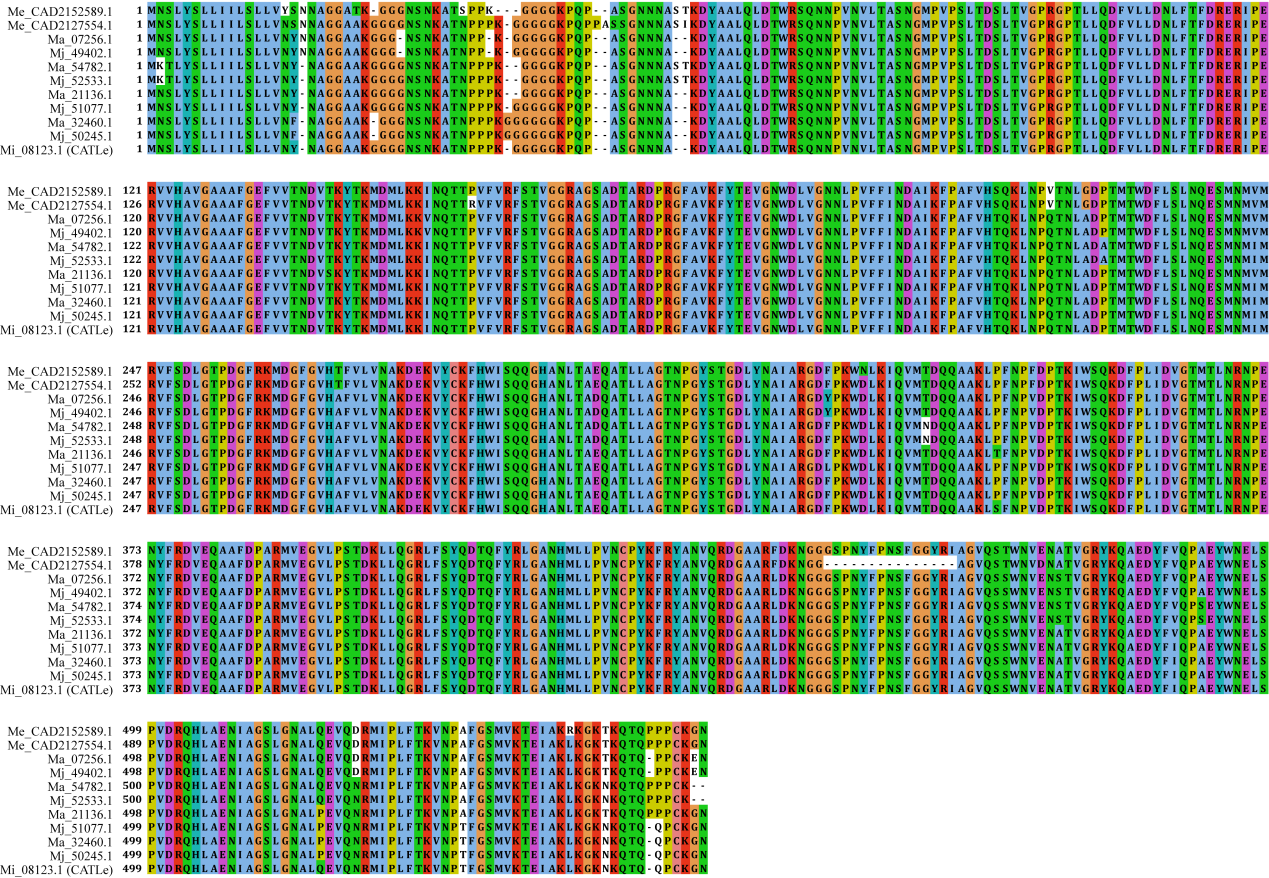


**Figure S1.** Multiple sequence alignment of proteins with secretory potential and homologous to CATLe from *M. enterolobii*, *M. arenaria*, and *M. javanica*.
