# Supplementary material for: Root‐knot nematodes exploit the catalase‐like effector to manipulate plant reactive oxygen species levels by directly degrading H2O2
Source: Mol Plant Pathol. 2024 Sep 10;25(9):e70000. doi: 10.1111/mpp.70000 (PMC11386320; doi:10.1111/mpp.70000)
Supplement: Supplementary file 2 — Figure S2. [file MPP-25-e70000-s005.docx]

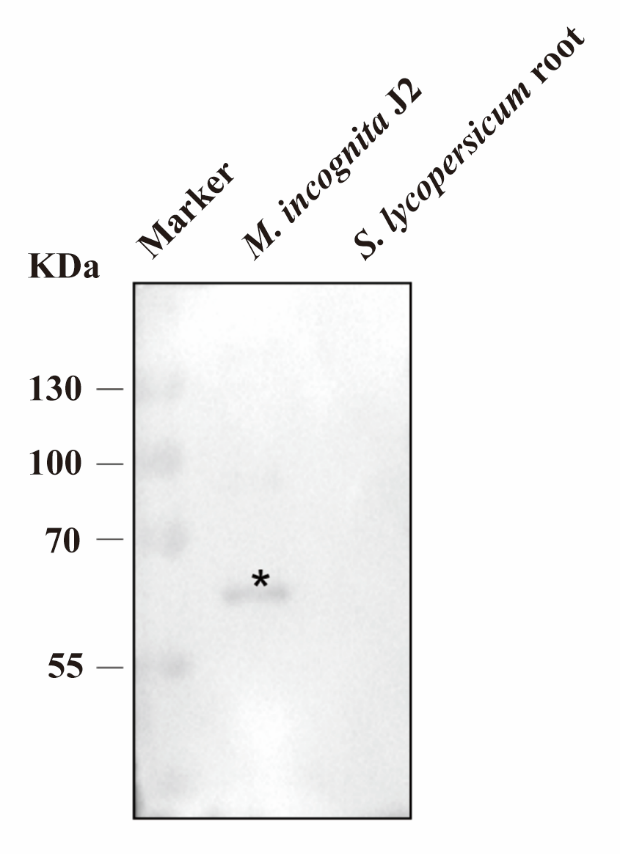


**Figure S****2.** Validation of anti-CATLe antibody specificity through Western blot. Immunodetection of CATLe by Western blot on total proteins from *M.incognita* J2 and from *S. lycopersicum* root. Western blot using CATLe antibody (1:5,000) and a goat anti-rabbit secondary antibodies coupled to HRP (1:10,000), * shows the CATLe at a size of ~62 KDa. Western blot also shows that CATLe antibody has no target protein in *S. lycopersicum* root.
