# Supplementary material for: Root‐knot nematodes exploit the catalase‐like effector to manipulate plant reactive oxygen species levels by directly degrading H2O2
Source: Mol Plant Pathol. 2024 Sep 10;25(9):e70000. doi: 10.1111/mpp.70000 (PMC11386320; doi:10.1111/mpp.70000)
Supplement: Supplementary file 3 — Figure S3. [file MPP-25-e70000-s004.docx]

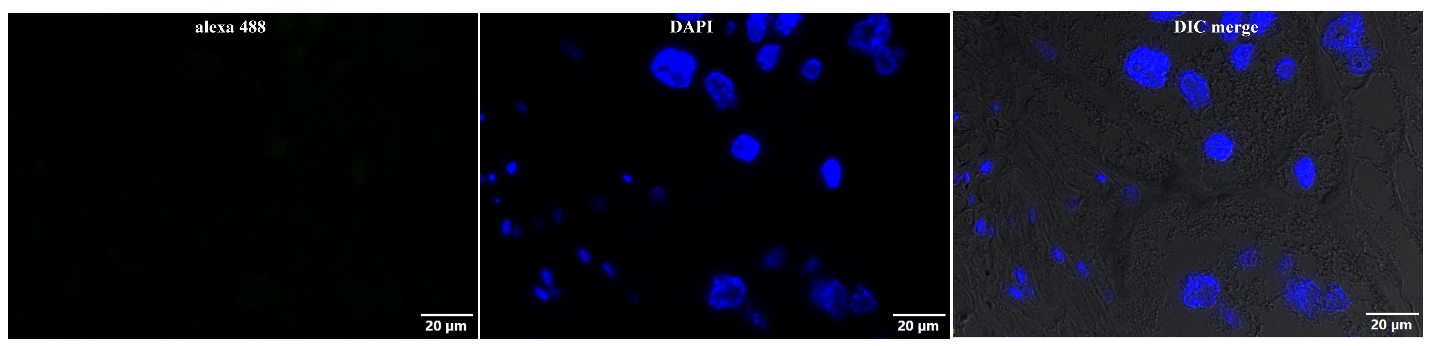


**Figure S3.** Immunolocalization of the secreted CATLe protein in plant tissues. The DIC images of *S. lycopersicum* root gall sections at 7 dpi treated with the pre-immune serum as a negative control. No fluorescence signal was detected within the giant cells. *, giant cells; Bar = 20 µm.
