# Supplementary material for: Root‐knot nematodes exploit the catalase‐like effector to manipulate plant reactive oxygen species levels by directly degrading H2O2
Source: Mol Plant Pathol. 2024 Sep 10;25(9):e70000. doi: 10.1111/mpp.70000 (PMC11386320; doi:10.1111/mpp.70000)
Supplement: Supplementary file 4 — Figure S4. [file MPP-25-e70000-s001.docx]

**
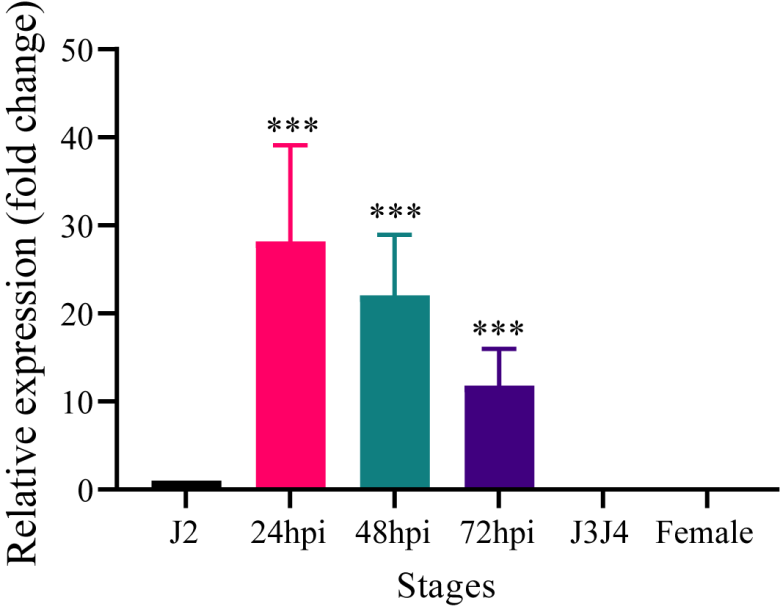
**

**Figure S4. qRT-PCR validation of the relative expression level of C-type lectin effector (Mi_30032, Mi_assembly_v1) in *M. incognita* at different infection stages.** The fold-change values were analyzed by the 2^-△△CT^ method. The *GADPH* gene of *M. incognita* was used as reference. *, *P* < 0.05, **, *P* < 0.01, ***, *P* < 0.001, Student's t test. Bars represent mean ± SD.
